# Deep learning system to predict the 5-year risk of high myopia using fundus imaging in children

**DOI:** 10.1038/s41746-023-00752-8

**Published:** 2023-01-26

**Authors:** Li Lian Foo, Gilbert Yong San Lim, Carla Lanca, Chee Wai Wong, Quan V. Hoang, Xiu Juan Zhang, Jason C. Yam, Leopold Schmetterer, Audrey Chia, Tien Yin Wong, Daniel S. W. Ting, Seang-Mei Saw, Marcus Ang

**Affiliations:** 1 Singapore National Eye Centre, Singapore Eye Research Institute, Singapore, Singapore; 2 Duke-NUS Medical School, National University of Singapore, Singapore, Singapore; 3 Escola Superior de Tecnologia da Saúde de Lisboa (ESTeSL), Instituto Politécnico de Lisboa, Lisboa, Portugal; 4 Comprehensive Health Research Center (CHRC), Escola Nacional de Saúde Pública, Universidade Nova de Lisboa, Lisboa, Portugal; 5 Asia Pacific Eye Centre, Gleneagles Hospital, Singapore, Singapore; 6 Yong Loo Lin School of Medicine, National University of Singapore, Singapore, Singapore; 7 Dept. of Ophthalmology, Columbia University, Columbia, SC USA; 8 Department of Ophthalmology and Visual Sciences, The Chinese University of Hong Kong, Hong Kong, China; 9 Hong Kong Eye Hospital, Hong Kong, China; 10 Department of Ophthalmology and Visual Sciences, Prince of Wales Hospital, Hong Kong, China; 11 Hong Kong Hub of Paediatric Excellence, The Chinese University of Hong Kong, Hong Kong, China; 12 Department of Ophthalmology, Hong Kong Children’s Hospital, Hong Kong, China

**Keywords:** Risk factors, Paediatric research

## Abstract

Our study aims to identify children at risk of developing high myopia for timely assessment and intervention, preventing myopia progression and complications in adulthood through the development of a deep learning system (DLS). Using a school-based cohort in Singapore comprising of 998 children (aged 6–12 years old), we train and perform primary validation of the DLS using 7456 baseline fundus images of 1878 eyes; with external validation using an independent test dataset of 821 baseline fundus images of 189 eyes together with clinical data (age, gender, race, parental myopia, and baseline spherical equivalent (SE)). We derive three distinct algorithms – image, clinical and mix (image + clinical) models to predict high myopia development (SE ≤ −6.00 diopter) during teenage years (5 years later, age 11–17). Model performance is evaluated using area under the receiver operating curve (AUC). Our image models (Primary dataset AUC 0.93–0.95; Test dataset 0.91–0.93), clinical models (Primary dataset AUC 0.90–0.97; Test dataset 0.93–0.94) and mixed (image + clinical) models (Primary dataset AUC 0.97; Test dataset 0.97–0.98) achieve clinically acceptable performance. The addition of 1 year SE progression variable has minimal impact on the DLS performance (clinical model AUC 0.98 versus 0.97 in primary dataset, 0.97 versus 0.94 in test dataset; mixed model AUC 0.99 versus 0.97 in primary dataset, 0.95 versus 0.98 in test dataset). Thus, our DLS allows prediction of the development of high myopia by teenage years amongst school-going children. This has potential utility as a clinical-decision support tool to identify “at-risk” children for early intervention.

## Introduction

Myopia is one of the leading causes of uncorrected, reversible visual impairment in the world^1–3^. It has been projected that myopia could affect 50% (4.7 billion) of the world’s population by 2050, with 10% (1 billion) suffering from high myopia^4,5^. High myopia carries an increased risk of sight-threatening complications such as retinal detachment, open-angle glaucoma, myopic macular degeneration, and choroidal neovascularization^6,7^ Occurrence of these complications would inevitably increase public health burden^8^, and lead to loss of productivity^9^.

Currently, known potential risk factors for the development of high myopia in children include a younger age of myopia onset^10–12^, higher myopia diagnosed at presentation^13^, rapid myopia progression^14,15^, reduced outdoor and increased near work time^14^, parental myopia^14^, education years^16^, and polygenic risk scores^17,18^. However, translating these factors into clinical practice can be challenging. Many of these factors are based on subjective recall, or require the use of cycloplegic eyedrops which may not be widely available due to the need for staff with higher level of training in administering the eyedrops^19,20^. Studies have attempted to develop risk prediction based on statistical^19,20^ approaches that rely on serial visual and refraction assessments, which is resource intensive and may delay necessary intervention^19^. Moreover, there is also increased demand for skilled eye care professionals, compounding challenges brought about by the growing magnitude of vision and eye health related problems^21^.

The trend of an increasing number of children and teenagers with high myopia has become a global concern^22^. This is particularly evident in East Asia where prevalence rates of high myopia have risen up to 21.6%^23–26^. While there are interventions such as atropine eyedrops and optical devices (e.g., myopic defocus spectacles and multifocal contact lenses) that may reduce myopia progression in children^27^, these interventions may not be suitable for all children and have potential side effects^28,29^.

A key area of research is to identify children who are “at risk” of developing high myopia with greater precision, so that these interventions may be appropriately introduced to these children. Thus, there is an unmet need to identify children at risk of developing high myopia, based on simple, accessible and objective measures, ideally at a single baseline visit. In this study, we aim to develop a deep learning system (DLS) utilizing objective fundus imaging and/or clinical data to identify children who are at risk of developing high myopia (5-year prediction) later in their teenage years.

## Results

From a total of 965 children (1878 eyes) with 7456 retina images from school 2 and 3, the deep learning model was trained with fivefold cross validation for detection of high myopia using 769 children (1502 eyes) with 5945 retina images and tested on 196 children (376 eyes) with 1511 retina images. The models were externally validated on the test dataset using 99 children (189 eyes) with 821 retina images from school 1. The overall subjects’ demographics, myopic status at baseline and in teenage years are listed in Supplementary Table 1. Comparison of baseline characteristics of subjects from the different schools showed that School 1 is generally statistically different from Schools 2 and 3 with the exception of proportion of males (*P* = 0.056) using Chi-squared test (Supplementary Table 2).

In the primary dataset for internal validation, among the 1502 eyes used in training and fivefold cross validation, 60.7% had no myopia, 29.8% had low myopia and 9.5% had moderate myopia at baseline. In teenage years, 5 years after the initial visit, 31.8% had no myopia, 36.9% had low myopia, 26.8% had moderate myopia and 4.6% had high myopia. In the 376 eyes used in testing, 62.2%, 28.7% and 9.0% had no, low, moderate myopia at baseline, respectively. After 5 years, 39.6%, 31.1%, 25.8% and 3.5% had no, low, moderate and high myopia, respectively. In the test dataset for external validation (189 eyes), 33.9%, 37.0% and 29.1% had no, low, moderate myopia at baseline, respectively. After 5 years, 23.3%, 33.9%, 34.4% and 8.5% had no, low, moderate and high myopia, respectively.

Using only variables at baseline, all our models achieved clinically acceptable performance. In the fundus image-only models, fundus image alone achieved an AUC of 0.93 in both the primary as well as the test dataset while in the clinical data-only models, baseline SE alone achieved an AUC of 0.90 and 0.93 in the primary and the test dataset, respectively. In the mixed models (image + clinical), the combination of fundus image and baseline SE achieved an AUC of 0.97 in both the primary as well as the test dataset. The addition of 1 year SE progression to the clinical and mixed models provided only marginal improvement or decline in model performance. The performance of the algorithm for image models, clinical models and mixed models is presented in Table 1.

**Table 1:** Internal and external validation of the DLS in image models, clinical models and mixed models for 5-year high myopia prediction.

Model	Input Variables	Internal Validation (School 2 and 3)					External Validation (School 1)				
		Threshold	AUC (C.I.)	Accuracy (C.I)	Sensitivity (C.I.)	Specificity (C.I.)	Threshold	AUC (C.I.)	Accuracy (C.I)	Sensitivity (C.I.)	Specificity (C.I.)
Image model	Fundus photo	1.94	0.93 (0.89–0.98)	0.84 (0.84–0.84)	0.92 (0.92–0.92)	0.84 (0.84–0.84)	1.94	0.93 (0.88–0.97)	0.86 (0.86–0.86)	0.88 (0.87–0.88)	0.86 (0.85–0.86)
	Fundus photo + Age + Race + Gender	1.84	0.95 (0.92–0.99)	0.86 (0.86–0.86)	0.85 (0.85–0.85)	0.86 (0.86–0.86)	1.84	0.91 (0.85–0.96)	0.78 (0.78–0.78)	0.88 (0.87–0.88)	0.77 (0.77–0.78)
Clinical model	Baseline SE^a^	1.74	0.90 (0.84–0.96)	0.80 (0.80–0.80)	0.77 (0.77–0.77)	0.80 (0.80–0.80)	1.74	0.93 (0.87–0.98)	0.65 (0.64–0.65)	1.00 (1.00–1.00)	0.61 (0.61–0.62)
	Baseline SE^a^ + Age + Race + Gender	1.99	0.97 (0.93–0.99)	0.87 (0.87–0.87)	0.85 (0.85–0.85)	0.87 (0.87–0.87)	1.99	0.94 (0.90–0.97)	0.74 (0.74–0.74)	1.00 (1.00–1.00)	0.72 (0.71–0.72)
	Baseline SE^a^ + Age + Race + Gender + 1-year SE* progression	2.06	0.98 (0.96–0.99)	0.92 (0.92–0.92)	0.92 (0.92–0.92)	0.92 (0.92–0.92)	2.06	0.97 (0.94–0.99)	0.84 (0.83–0.84)	1.00 (1.00–1.00)	0.82 (0.82–0.82)
Mixed model	Fundus photo + Baseline SE^a^	1.98	0.97 (0.94–0.99)	0.93 (0.93–0.93)	0.92 (0.92–0.92)	0.93 (0.93–0.93)	1.98	0.97 (0.94–0.99)	0.82 (0.82–0.82)	1.00 (1.00–1.00)	0.80 (0.80–0.81)
	Fundus photo + Baseline SE^a^ + Age + Race + Gender	1.98	0.97 (0.95–0.99)	0.93 (0.93–0.93)	0.92 (0.92–0.92)	0.93 (0.93–0.93)	1.98	0.98 (0.96–1.00)	0.82 (0.82–0.82)	1.00 (1.00–1.00)	0.80 (0.80–0.81)
	Fundus photo + Baseline SE^a^ + Age + Race + Gender + 1-year SE^a^ progression	2	0.99 (0.97–1.00)	0.94 (0.94–0.94)	0.92 (0.92–0.92)	0.94 (0.94–0.94)	2	0.95 (0.92–0.98)	0.88 (0.88–0.88)	0.94 (0.94–0.94)	0.88 (0.88–0.88)

*AUC* area-under-curves, *C.I.* confidence interval.

^a^Spherical equivalent (SE) was performed using cycloplegic autorefraction.

### Fundus image-only models

**Fig. 1:**
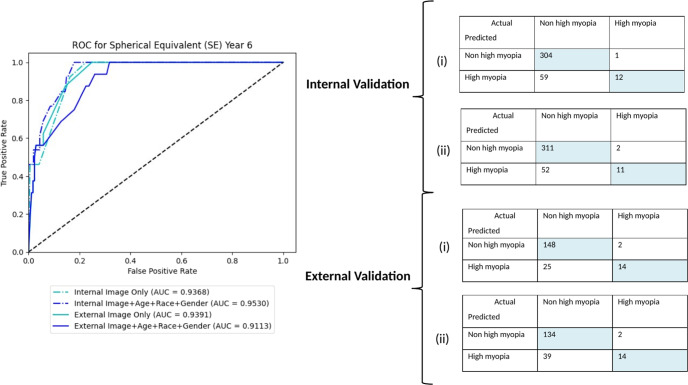
Performance of image models. AUC and confusion matrixes for internal and external validation of image models—(i) Fundus photo only and (ii) Fundus photo + Age + Race + Gender.

The algorithm with baseline childhood fundus imaging input alone achieved clinically acceptable prediction of high myopia in teenage years. The AUC and accuracy in classification was 0.93, 0.84 and 0.93, 0.86 for internal and external validation, respectively. The addition of age, race and gender resulted in marginal improvement or decline in performance (AUC 0.95 in primary dataset, 0.91 in test dataset). The AUC curves and confusion matrixes for internal and external validation are shown in Fig. 1.

### Clinical data-only models

**Fig. 2:**
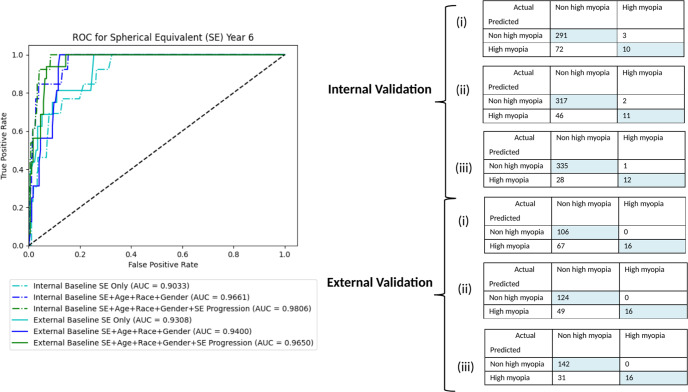
Performance of clinical models. AUC and confusion matrixes for internal and external validation of clinical models – (i) Baseline SE, (ii) Baseline SE + Age + Race + Gender, and (iii) Baseline SE + Age + Race + Gender + 1-year SE progression.

Two baseline clinical models were developed for (i) Baseline SE, (ii) Age + Race + Gender + Baseline SE. The AUC and accuracy achieved was 0.90–0.97, 0.80–0.87 and 0.93–0.94, 0.65–0.74 for internal and external validation, respectively. Similar to the fundus image-only model, additional input with 1 year progression data led to marginal improvement in performance (AUC 0.98 in primary dataset, 0.97 in test dataset). The addition of parental myopia did not further improve the performance. The AUC and confusion matrixes for internal and external validation are shown in Fig. 2, while the random forest feature importance for clinical models can be found in Supplementary Fig. 1.

### Mixed Models

**Fig. 3:**
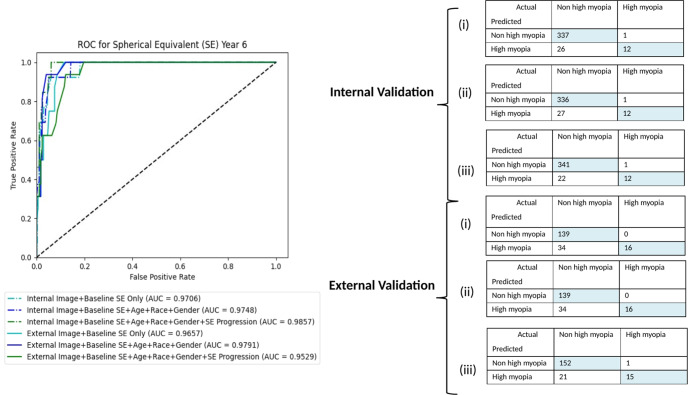
Performance of mixed models. AUC and confusion matrixes for internal and external validation of mixed models—(i) Fundus photo + Baseline SE, (ii) Fundus photo + Baseline SE + Age + Race + Gender, and (iii) Fundus photo + Baseline SE + Age + Race + Gender + 1-year SE progression.

The algorithm with baseline childhood fundus imaging together with clinical data input achieved the highest performance in prediction of high myopia in teenagers. Two baseline mixed models were developed for (i) Fundus photo + Baseline SE and (ii) Fundus photo + Age + Race + Gender + Baseline SE with AUC and accuracy in classification of 0.97, 0.93 and 0.97–0.98, 0.82 for internal and external validation, respectively. The addition of 1 year progression input data delivered marginal improvement or decline in performance (AUC 0.99 in primary dataset, 0.95 in test dataset). Similar to the clinical model, the addition of parental myopia did not further improve the performance. The AUC and confusion matrixes are shown in Fig. 3, respectively- The random forest feature importance for mixed models can be found in Supplementary Fig. 1.

### Performance of models for AL prediction

We have also attempted to perform long axial length (AL ≥ 26.5mm) prediction in image models, clinical models and mixed models. In the primary dataset for internal validation, among the 1502 eyes used in training and fivefold cross validation, 100% had AL < 26.5mm at baseline. In teenage years, five years after the initial visit, 98.1% had AL < 26.5mm while 1.86% (28 eyes) had AL ≥ 26.5 mm. In the 376 eyes used in testing, 100% had AL <26.5 mm at baseline and 2.13% (8 eyes) had AL ≥ 26.5 mm after 5 years. In the test dataset for external validation (189 eyes), 100% had AL <26.5 mm at baseline and 8.47% (16 eyes) had AL ≥ 26.5 mm after five years (Supplementary Table 1).

In the fundus image-only models, fundus image alone achieved an AUC of 0.67 and 0.57 while in the clinical data-only models, baseline AL alone achieved an AUC of 0.83 and 0.91 in the primary and the test dataset, respectively. In the mixed models (image + clinical), the combination of fundus image and baseline AL achieved an AUC of 0.98 and 0.88 in the primary and the test dataset, respectively. The addition of 1 year SE progression to the clinical and mixed models provided only marginal or no improvement in model performance. The performance of the algorithm for image models, clinical models and mixed models were presented in Table 2.

**Table 2:** Internal and external validation of the DLS in image models, clinical models and mixed models for 5-year axial length ≥26.5 mm prediction.

Model	Input Variables	Internal Validation (School 2 and 3)					External Validation (School 1)				
		Threshold	AUC (C.I.)	Accuracy (C.I)	Sensitivity (C.I.)	Specificity (C.I.)	Threshold	AUC (C.I.)	Accuracy (C.I)	Sensitivity (C.I.)	Specificity (C.I.)
Image model	Fundus photo	0	0.67 (0.48–0.87)	0.94 (0.94–0.94)	0.38 (0.37–0.38)	0.95 (0.95–0.95)	0	0.57 (0.47–0.69)	0.84 (0.84–0.84)	0.25 (0.25–0.25)	0.90 (0.90–0.90)
	Fundus photo + Age + Race + Gender	0.03	0.86 (0.76–0.96)	0.77 (0.77–0.77)	0.63 (0.62–0.63)	0.77 (0.77–0.77)	0.03	0.64 (0.49–0.80)	0.69 (0.69–0.70)	0.63 (0.62–0.63)	0.70 (0.70–0.70)
Clinical model	Baseline AL^a^	0	0.83 (0.62–0.98)	0.84 (0.84–0.84)	0.75 (0.75–0.75)	0.84 (0.84–0.84)	0	0.91 (0.86–0.96)	0.67 (0.67–0.67)	1.00 (1.00–1.00)	0.64 (0.64–0.64)
	Baseline AL^a^ + Age + Race + Gender	0.01	0.93 (0.89–0.97)	0.86 (0.86–0.86)	0.75 (0.75–0.75)	0.86 (0.86–0.86)	0.01	0.95 (0.92–0.98)	0.71 (0.71–0.72)	1.00 (1.00–1.00)	0.69 (0.69–0.69)
	Baseline AL^a^ + Age + Race + Gender + 1-year AL* progression	0.04	0.95 (0.91–0.98)	0.88 (0.87–0.88)	0.88 (0.87–0.88)	0.88 (0.87–0.88)	0.04	0.94 (0.90–0.98)	0.74 (0.74–0.74)	0.94 (0.94–0.94)	0.72 (0.72–0.72)
Mixed model	Fundus photo + Baseline AL^a^	0.17	0.98 (0.95–1.00)	0.93 (0.93–0.93)	0.88 (0.87–0.88)	0.93 (0.93–0.94)	0.17	0.88 (0.83–0.93)	0.85 (0.85–0.85)	0.75 (0.75–0.75)	0.86 (0.85–0.86)
	Fundus photo + Baseline AL^a^ + Age + Race + Gender	0.07	0.98 (0.96–1.00)	0.94 (0.94–0.94)	1.00 (1.00–1.00)	0.94 (0.94–0.94)	0.07	0.89 (0.83–0.94)	0.86 (0.86–0.86)	0.81 (0.81–0.81)	0.87 (0.87–0.87)
	Fundus photo + Baseline AL^a^ + Age + Race + Gender + 1-year AL^a^ progression	0.16	0.98 (0.96–1.00)	0.92 (0.92–0.92)	0.88 (0.87–0.88)	0.92 (0.92–0.92)	0.16	0.90 (0.84–0.94)	0.83 (0.82–0.83)	0.69 (0.69–0.69)	0.84 (0.84–0.84)

*AUC* area-under-curves, *C.I.* confidence interval.

^a^Axial length (AL) was performed using contact ultrasound biometry.

## Discussion

In this study, we developed a modular DLS based on single-time point objective data and fundus imaging to predict the 5-year development of high myopia in a multi-ethnic group of children aged between 6 and 12 years old at baseline. Our models demonstrated clinically acceptable predictive performance with AUCs ranging from 0.90 to 0.98. Importantly, the fundus image-only model demonstrated comparable performance (AUC = 0.94) against clinical models (AUC 0.90–0.97). Marginal benefit or decline in performance was noticed with additional 1 year follow-up progression data of SE. The performance of our fundus image-only model has the potential to be translated and implemented into community or school-based programs to identify at-risk children for further assessment and intervention if required.

In the recent report *Impact of Myopia* by the International Myopia Institute (IMI), the global cost of myopia care is expected to increase from an estimated USD$358.7 billion in 2019 to USD$ 870 billion by 2050^30^. In particular, these reports indicate that high and pathologic myopia place significant financial burden on both the individual and society, with annual costs increasing substantially with age^30^. Thus, the IMI *Clinical Management Guidelines Report* advocates for myopia control, which includes risk assessment, followed by clinical evaluation, and treatment selection^31^. In addition, identifying children “at risk” of high myopia and those with “premyopia” are important for early intervention^32^. Though such broad definitions could support clinical decision making, in reality, accurately identifying a child at risk of developing high myopia in clinical practice is challenging. High-risk features such as family history^33–36^ and environmental factors (near work and outdoor exposure)^37–41^ are helpful but not deterministic, hence current childhood myopia management is heavily reliant on eyecare professionals’ judgment and experience. Based on the current available clinical evidence^42–48^, children who are already myopic may be started on myopia control therapies to prevent further progression; while environmental and behavioral modifications should be encouraged in ‘pre-myopic’ high-risk children. However, current clinical management requires a more precise approach that is individualized, to reliably identify and initiate timely treatment for high-risk children.

Our predictive DLS is designed to address these specific challenges, and with the most clinical impact. First, we have a target age group of children aged 6–12 years old who are most vulnerable to myopia progression and also amenable to myopia control therapies^31,49^. Second, we used only objective inputs to avoid biases related to subjective recall—and these are obtained at one single time point (baseline), thereby eliminating the need for repeated, longitudinal follow-up before a clinical decision can be made and avoiding unnecessary delay of treatment for high-risk individuals. Third, our various image-based and mixed-clinical models produced clinically acceptable performances, allowing for implementation in various clinical settings with the availability of imaging systems. As a further enhancement, our DLS was trained and tested using a multi-ethnic population which improves the overall generalizability of the results. Fourth, our DLS delivered robust predictive performance against an external validation dataset which was dissimilar to the training datasets. This suggests that our DLS could have the capacity to achieve good performance against unique external datasets, which would require substantiation through further validations.

Comparing our fundus image-only model with other models in this study that require clinical predictors input, using baseline fundus image alone appeared to be comparable in performance and adequate in predicting 5-year high myopia. We also compared our fundus image-only model to previous studies using regression methods^19^ and machine learning (ML)^20^ to predict high myopia in childhood (Supplementary Table 3). Our approach provided several distinct advantages. Firstly, our DLS based baseline fundus image as a single input variable, was able to exceed or was at least on par with the performance of the 5-year high myopia prediction ML algorithms, using big data (age and refraction) from electronic medical records, proposed by Lin et al. However, the ML algorithms required cycloplegic refraction and a minimum of three repeated annual visits before a prediction could be made^20^. Secondly, the statistical models in Chen et al were only able to predict the development of high myopia at 18 years old (5 or 6-year prediction) in 12 to 13 years old children, using age, gender and cycloplegic SE ranging from 1–3 visits^19^. However, by 18 years of age, myopia progression and axial elongation would have occurred, missing the window period for myopia control treatment. In comparison, our models target children aged 6–12 years old in order for potential myopia interventions to remain effective. Moreover, our DLS was also able to achieve comparable performance utilizing the same variables used in models proposed by Chen et al. (Supplementary Table 3).

Logistically, utilizing baseline fundus image alone can eliminate the need for cycloplegic refraction^19^ without significant degradation in predictive performance. Cycloplegic refraction is a time consuming process with a waiting time ranging between 1 and 2 h. Additionally, the children routinely experience side effects of pupil dilation and glare lasting up to 72 h. Hence, this procedure is not routinely performed in clinical assessments or myopia screening programs^50^. On the other hand, predictive utility of non-cycloplegic SE alternatives such as manifest refraction or basic autorefraction could be confounded by the spurious effects of pseudomyopia. In comparison, our approach utilizing fundal imaging coincides with the maturation of non-mydriatic imaging technology. It is now feasible to obtain high quality images with minimal latency. This provides comparative advantages, including significant time savings versus cycloplegic SE and better accuracy versus non-cycloplegic SE modalities.

The implementation of AI into myopia screening or evaluation programs will depend on the availability of skilled manpower, imaging systems and infrastructure support, and therefore the selection (or design) of a candidate system needs to consider these issues^51–54^. The World Report on Vision highlighted the increasing demand for trained eye health human resources to address the substantial burden of vision and eye health issues globally^55^. With 19 million children with visual impairment, including blindness in 1.4 million and low vision in 17.5 million^56^, the shortage of trained eye care professionals particularly optometrists and ophthalmologists, represent major barriers to accessing quality childhood myopia management^21^. Majority of these children reside in developing countries^56^ which are chronically underserved due to a paucity of optometrists and unequal optometrist workforce distribution^57^. This gap would likely widen with time, given the increasing trend in economic inequality.

For example, our image-only model based on a single baseline fundus photograph may be implemented easily without the need for other sophisticated, costly equipment to measure AL and autorefraction. It also eliminates the need to perform cycloplegic refraction which requires skilled manpower and is difficult to conduct in primary healthcare settings^58^. In the future, such DLS image analysis could be integrated into the fundus camera or performed using cloud computing^59^. Separately, our DLS system may be integrated into national myopia screening programs^60,61^, or even in less developed healthcare systems that have access to portable fundal cameras^62^. However, this would need to be balanced against the higher false-negative rate in comparison with models solely based on clinical data or a mixture of both. This may result in misclassification of children at-risk, resulting in the subsequent development of high myopia. It is therefore important for public health experts and policies makers to weigh the risk of screening inaccuracies against the benefit of scalable large-scale screening based on a single fundus photograph. In addition, incidences of misclassification may also be minimized through regular annual screenings. On the other hand, the mixed-model would be better suited to a tertiary healthcare setting where imaging equipment and cycloplegic refraction are readily available. This would then function as a clinical-decision support tool by identifying suitable candidates for treatment.

**Fig. 4:**
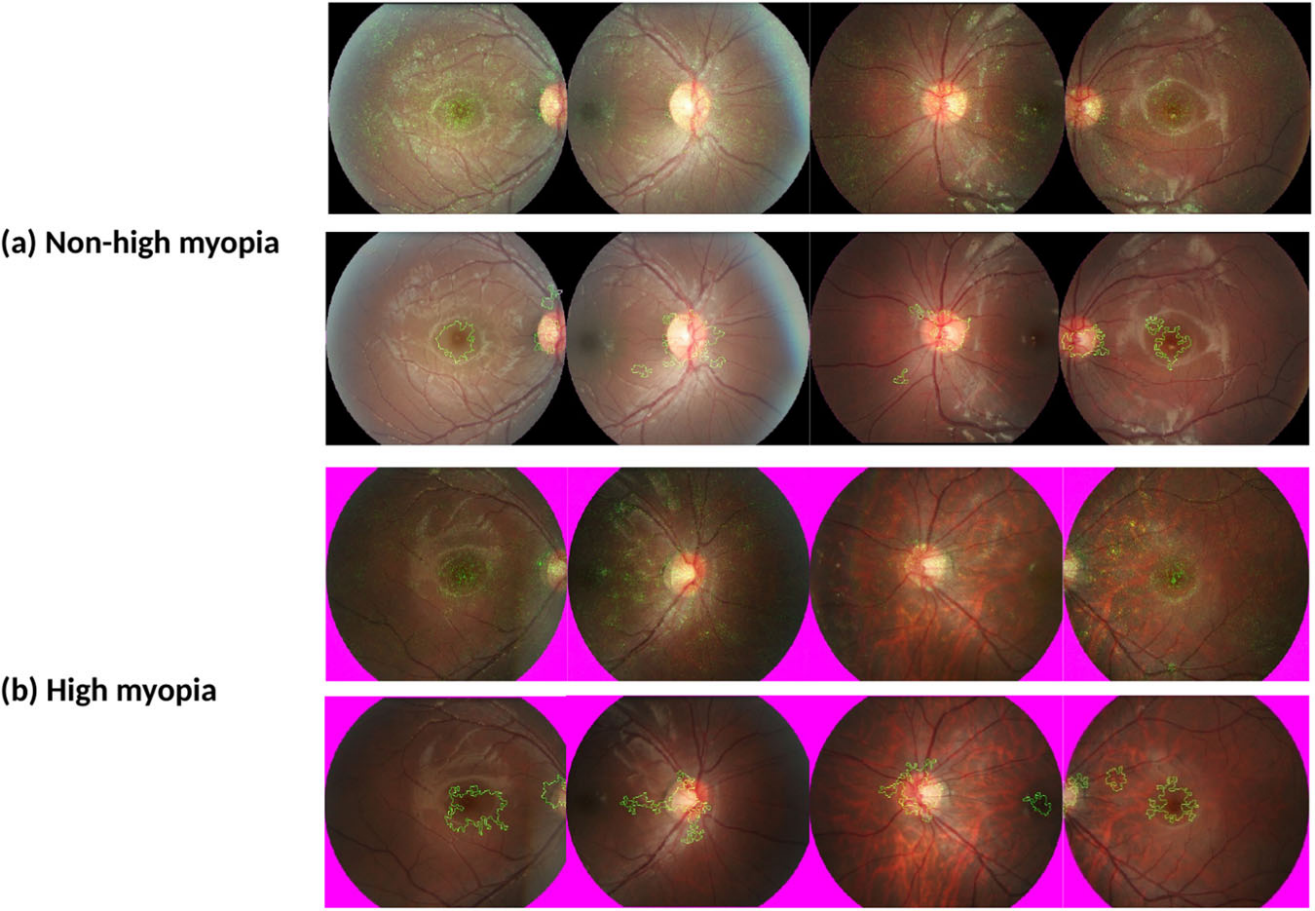
Saliency heatmaps. Integrated Gradients technique (Top) and post-processed images demonstrating areas of interest through collation of high saliency pixels (Bottom) for **a** Non-high myopia and **b** High myopia.

In order to identify at risk individuals, we postulate that our DLS could have indirectly detected the early phases of accelerated myopia development through higher presenting SE as well as subtle morphological changes on fundus imaging. This could include differences in macular choroidal thickness or topographical differences at the macular and disc. Moreover, post-processing techniques applied to saliency heatmaps, generated using Integrated Gradient techniques, had identified the disc and macular as areas of interest (Fig. 4), consistent with areas of future myopic disc changes and myopic macular degeneration^63^.

Our study has several limitations. Firstly the distribution of children with and without high myopia in our current cohort after 5 years exhibited significant imbalance. While this could affect the utility of our results, it would be an unavoidable challenge as our dataset is reflective of the naturally skewed-distribution of the disease. Furthermore, with Singapore ranking amongst the highest in the world in terms of high myopia prevalence, our current data distribution is likely to reflect the upper end of high myopia disease frequency in the real world. Hence, augmenting with population data from other studies would represent an impractical effort to address this constraint.

While our algorithm was developed using a longitudinal school-based dataset, the algorithm would require further testing in other cohorts with different study population. Ideally, external validation involving children of different ethnicity and geographical locations could be performed—but similar longitudinal cohorts that capture fundus images from childhood and with 5-year longitudinal follow up, are not widely available. Furthermore, it is challenging to locate treatment naïve longitudinal cohorts in children for external validation, due to ethical considerations in withholding treatment. To address this, we are in active engagements with international partners to create a consortium to collate required data prospectively in the future.

Next, fundal images were obtained by a single mydriatic fundus camera platform (CR6-NM45, EOS-D60, Cannon) under cycloplegic conditions. However, with the introduction of non-mydriatic fundus cameras, fundus image capture is now more widely available without the need for application of eyedrops for pupil dilation. Thus, further studies are required to assess the performance of our DLS across various fundus image systems including non-mydriatic fundus cameras. However, it should be noted that studies have established that the performance of automated AI analysis between non-mydriatic and mydriatic cameras platforms for screening of undiagnosed diabetic retinopathy has been shown to be highly comparable^64^.

In addition, we were unable to achieve clinically acceptable performance in AI prediction for AL, particularly in fundus image-only model due to the small dataset available for training and testing with limited numbers of subjects with AL ≥ 26.5mm at 5-years (internal validation = 36 eyes, external validation = 16 eyes). Further studies with larger datasets would be required to assess the performance of AL prediction.

In summary, we have developed a DLS using a baseline fundus image and objective clinical data (age, race, gender, baseline SE) to identify schoolchildren at risk of developing high myopia later on in their teenage years. Through early identification, targeted and timely myopia control therapies may be instituted to reduce the risk of developing high myopia in these children. The fundus image-only model may be implemented via integration into fundus camera systems or cloud-based computing. However, further external validation in various treatment naïve populations and using new non-mydriatic cameras will further strengthen the potential application of this AI system. Nonetheless, we present promising results from a DLS that addresses several clinical challenges faced by myopia evaluation and prevention programs, by reducing reliance on cycloplegic refraction, axial length measurements or repeated reviews. With further development of this AI system, it may be used as a clinical assistive tool to identify children “at risk” of developing high myopia with greater precision and introduce myopia control therapies if needed.

## Methods

### Study design

In this retrospective population-based study, high myopia was defined as spherical equivalent (SE) ≤ −6 D or axial length (AL) ≥ 26.5 mm^15,32^. We predicted the development of each class using fundus images and/or clinical data. Three types of models were developed—image-only, clinical data-only and mixed (clinical + image). The models were trained, validated and tested using the Singapore Cohort of Risk factors for Myopia (SCORM) dataset^65–67^.

The image-only models were developed using pre-processed fundus images and pre-trained DenseNet-121 deep neural network models. These image models were then used to generate image-based risk scores for each eye. The clinical data-only models utilized random forest to extract the relevant clinical features and generate a clinical data-based risk score for each eye. For the mixed model, the image-based scores, which represented clinical features extracted from image data, were combined with the clinical data-based scores to derive an overall mixed-model risk score.

The study was approved by the Ethics Committee at the Singapore Eye Research Institute and the Centralized Institutional Review Boards of the Singapore Health Services (2016/2215) and conducted in accordance with the tenets of the Declaration of Helsinki. Written informed consent was obtained from the parents after the nature of the study was explained.

### Clinical training, validation, and testing datasets

**Fig. 5:**
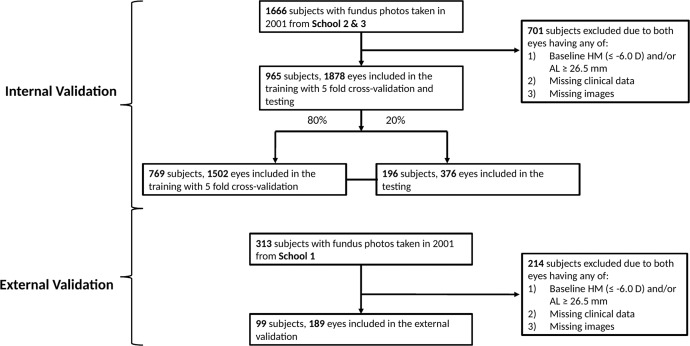
Flowchart of dataset. Singapore Cohort of Risk factors for Myopia was used in Deep Learning System training/internal validation (School 2 and 3) and external validation (School 1).

In the SCORM study cohort, children from grades 1 to 3 were recruited from three Singapore schools (*n* = 1979) based on methodology previously described^65–67^. The exclusion criteria included children with serious medical conditions or syndromes associated with myopia or any eye disorders at baseline. Questionnaires in the three most common languages (English, Chinese, and Malay) were administered to parents by a trained interviewer during the baseline visit. This was performed to obtain demographic data, including the number of parents with myopia^65^. Parents were considered myopic if they required corrective lenses for distance vision. Data for this study was derived from 1979 children (aged 6–12 years), who attended the visit in 2001 (baseline), 2002 (1-year follow up) and 2006 (5-year follow up) visits. Fundus imaging was only performed at 2001 (baseline). None of these children had myopia control treatment during the follow-up period. The primary dataset comprised of 1666 subjects from schools 2 and 3, of which 701 subjects were excluded due to baseline high myopia and/or missing data (clinical/fundus image). For the primary validation, 965 subjects (1878 eyes) with 7456 retina images were included in training/validation and testing of AI algorithm. The training/validation to testing data set was split randomly using a 4:1 ratio, with 769 subjects (1502 eyes) in the training set and 196 subjects (376 eyes) in the test set (Fig. 5). The independent test dataset comprised of 313 subjects from schools 1, of which 214 subjects were excluded due to baseline high myopia and/or missing data (clinical/fundus image). For the external validation, 99 subjects (189 eyes) with 821 retina images were included.

### Eye measurements and imaging

Annual cycloplegic refraction was performed for the participants. One drop of topical proparacaine 0.5% was first instilled followed by three drops of 1% cyclopentolate instilled at 5 min intervals to achieve sufficient cycloplegic response. Cycloplegic autorefraction was then performed after an interval of at least 30 min after the last eye drop. This was performed using a table-mounted autorefractor (model RK5; Canon, Japan). In total, five measurements were performed per eye (ensuring maximum difference between readings were <0.25 D apart) and total mean was used for analysis. AL measurements were obtained using contact ultrasound biometry (Echoscan model US-800, probe frequency 10 mHz; Nidek Co., Ltd., Tokyo Japan) after instillation of 1 drop of 0.5% proparacaine. The average of six measurements was taken and accepted only if the SD of these readings was less than 0.12 mm. Subsequently, the SE for each eye was calculated based on the formula sphere power plus half cylinder power. After pupil dilatation, digital retinal photographs centered on the optic disc were taken for both eyes using standardized settings (6.3 mega-pixel, resolution 3072 × 2048; CR6-NM45, EOS-D60; Canon USA, Lake Success, NY). Myopia was defined as an SE ≤ −0.5 D with low myopia (-3.0 D < SE ≤ −0.5 D), moderate myopia (−6.0 D < SE ≤ −3.0 D) and high myopia (SE ≤ −6 D). One-year mean SE and AL progression were calculated as SE at year 2002 visit minus SE at year 2001 (baseline) and AL at year 2002 visit minus AL at year 2001 (baseline), respectively.

### Architecture of deep learning system

We utilized baseline childhood fundus photographs as the input for the development of the image-only models. All fundus photographs were first pre-processed into a square template to extract the central circular region, which then underwent contrast normalization. Pre-trained DenseNet-121 deep neural network models were employed, with a batch size of 16, an initial learning rate of 0.001, Nesterov momentum of 0.90 and categorical cross-entropy loss. In addition, image augmentation procedures were used, as follows, in order to increase the dataset size and to strengthen model generalizability: (1) rotation (clockwise by 0–180°, selected randomly), (2) horizontal flip, and (3) vertical flip. Brightness and scale adjustment were also performed for training inputs. Local contrast normalization of the retinal fundus photos took place after the central disc of the photo had been extracted to a template image of 512 × 512 pixels. The Contrast Limited Adaptive Histogram Equalization (CLAHE) method was then applied with a kernel size of 51 pixels. These networks were trained with two output nodes corresponding to the two target classes for SE (SE ≤ −6 D or >−6 D) and AL (AL > 26.5 mm or ≥ 26.5 mm). Training was performed until convergence on accuracy was demonstrated with the internal validation data. An early stop procedure was applied to avoid overfitting: the training was stopped if the loss on the validation set no longer decreased for 5 epochs.

Saliency heatmaps were generated on the template images by employing the Integrated Gradients technique^68^. The Integrated Gradients technique has the advantage of fulfilling theoretical sensitivity and implementation invariance axioms by design, and being able to generate pixel-level saliency estimates, unlike other popular visualization methods. The heatmaps were subsequently post-processed by applying thresholding techniques to the Integrated Gradients pixel-level outputs, thereby emphasizing the pixels with the highest saliency. This is followed by the application of an image morphology operation to collate these high-saliency pixels into larger representative regions.

Development of the clinical data-only models involved extraction of the relevant clinical feature scores for each eye. Subsequently, grid search with five-fold cross-validation was used to determine the optimal random forest hyperparameter values for each model. The hyperparameters were optimized for the number of estimators, the maximum number of features, the maximum depth and the split quality criterion. Once the optimal hyperparameter values were determined, the actual random forest model was trained on the entire training data.

Preliminary experiments had been attempted with using feature vectors extracted just before the output node layer, of 10 and 100 nodes, respectively. The final random forest model utilizing just the output node values generally outperformed models utilizing a larger number of features extracted from previous intermediate layers. As such, the output node layer values were used to develop the actual experimental models as described below.

From the trained image-only models, image-based scores were generated for each eye. All images for an eye were first processed by the model, to produce an image-level score. These image-level scores were calculated from the individual output node value, by multiplying the value of each node with the index of the node (which corresponds to the severity of the condition), and then summing these values into a single representative score for that condition. In predicting myopia severity, the corresponding image model would predict four separate probabilities at its four output nodes, including the development of no myopia (node index 0), low myopia (node index 1), moderate myopia (node index 2) and high myopia (node index 3), respectively. These four probabilities were constrained to add up to the value of 1, with the use of the softmax function. To convert these four probabilities into a single value for the final predicted severity of myopia, each individual probability were multiplied by the node index and then added together. For example, if an image was predicted to have no myopia with a probability of 1, its image-level score would be 0 (1 × 0). If an image was predicted to have moderate myopia with a probability of 0.5 and high myopia with a probability of 0.5, the resulting image-level score would be 2.5 (0.5 × 2 + 0.5 × 3). Next, the eye-level score was computed as the average of all image-level scores from the images of that particular eye. The resulting eye-level scores can then be considered as a clinical feature that is automatically obtained from image data. These scores were subsequently integrated with the clinical data-only models, in the same manner as other clinical features, during the development of mixed models.

### Statistical analysis

For each of the AI algorithms for detection of high myopia against ground truths, we calculated the area under the receiver operating characteristic (ROC) curve (AUC), accuracy, sensitivity and specificity for each classification threshold applied to the validation datasets. A classification threshold to achieve pre-determined sensitivity and specificity of at least 75% was set. The algorithms were tested on the independent testing datasets on different AI models—1) fundus image-only, 2) clinical data-only and 3) mixed model, with the previously-found output thresholds applied as operating points. For computing confidence interval estimation, bootstrapping was used only to estimate 95% confidence interval (CI) for the performance metrics of our classification results (i.e., AUC, sensitivity, specificity and accuracy). We applied n-out-of-n bootstrap with replacement at eye level from our dataset. For each bootstrap sample, we calculated and reserved the performance metrics for that bootstrap sample. The bootstrap sampling was repeated for 1000 times. We then estimated the 95% CI by using the 2.5 and 97.5 percentiles of the empirical distribution of corresponding metrics. Confusion matrices were used to assess the differences in classification performance for each model with the row and column representing predicted and true results, respectively. Comparison of baseline characteristics amongst the three schools was performed using analysis of variance and Chi-squared test for continuous and categorical variables, respectively. *P* values < 0.05 were considered statistically significant. All statistical analyses were performed using Python version 3.6.8 and SciPy version 1.5.4.

### Reporting summary

Further information on research design is available in the Nature Research Reporting Summary linked to this article.

## Supplementary information

Reporting Summary

Supplementary Material

## Data Availability

The data that support the findings of this study are available from the corresponding author, [MA], upon reasonable request.

## Code availability

The algorithms of this study are available for download at: https://www.dropbox.com/s/n6nsmia4gwjghka/SCORM_code.zip?dl=0.
