# Supplementary material for: Deep learning system to predict the 5-year risk of high myopia using fundus imaging in children
Source: NPJ Digit Med. 2023 Jan 26;6:10. doi: 10.1038/s41746-023-00752-8 (PMC9879938; doi:10.1038/s41746-023-00752-8)
Supplement: Supplementary file 1 — Reporting Summary [file 41746_2023_752_MOESM1_ESM.pdf]

nature portfolio

Corresponding author(s): Marcus Ang

Last updated by author(s): Dec 22, 2022

Reporting Summary

Nature Portfolio wishes to improve the reproducibility of the work that we publish. This form provides structure for consistency and transparency in reporting. For further information on Nature Portfolio policies, see our Editorial Policies and the Editorial Policy Checklist.

Statistics

For all statistical analyses, confirm that the following items are present in the figure legend, table legend, main text, or Methods section.

n/a	Confirmed	
☐	☒	The exact sample size (*n*) for each experimental group/condition, given as a discrete number and unit of measurement
☐	☒	A statement on whether measurements were taken from distinct samples or whether the same sample was measured repeatedly
☐	☒	The statistical test(s) used AND whether they are one- or two-sided<br>*Only common tests should be described solely by name; describe more complex techniques in the Methods section.*
☐	☒	A description of all covariates tested
☐	☒	A description of any assumptions or corrections, such as tests of normality and adjustment for multiple comparisons
☐	☒	A full description of the statistical parameters including central tendency (e.g. means) or other basic estimates (e.g. regression coefficient) AND variation (e.g. standard deviation) or associated estimates of uncertainty (e.g. confidence intervals)
☐	☒	For null hypothesis testing, the test statistic (e.g. *F*, *t*, *r*) with confidence intervals, effect sizes, degrees of freedom and *P* value noted<br>*Give P values as exact values whenever suitable.*
☒	☐	For Bayesian analysis, information on the choice of priors and Markov chain Monte Carlo settings
☒	☐	For hierarchical and complex designs, identification of the appropriate level for tests and full reporting of outcomes
☒	☐	Estimates of effect sizes (e.g. Cohen's *d*, Pearson's *r*), indicating how they were calculated

Our web collection on statistics for biologists contains articles on many of the points above.

Software and code

Policy information about availability of computer code

Data collection	No software was used
Data analysis	Python version 3.6.8 and SciPy version 1.5.4.

For manuscripts utilizing custom algorithms or software that are central to the research but not yet described in published literature, software must be made available to editors and reviewers. We strongly encourage code deposition in a community repository (e.g. GitHub). See the Nature Portfolio guidelines for submitting code & software for further information.

Data

Policy information about availability of data

All manuscripts must include a data availability statement. This statement should provide the following information, where applicable:

- Accession codes, unique identifiers, or web links for publicly available datasets
- A description of any restrictions on data availability
- For clinical datasets or third party data, please ensure that the statement adheres to our policy

The data that support the findings of this study are available from the corresponding author, [MA], upon reasonable request. The algorithms of this study are available for download at: https://www.dropbox.com/s/n6nsmia4gwjghka/SCORM_code.zip?dl=0

Human research participants

Policy information about studies involving human research participants and Sex and Gender in Research.

Reporting on sex and gender	In this study, sex (biological attribute) data had been assigned and collected from the Singapore Cohort Study of the Risk Factors of Myopia (SCORM) study was used in the algorithm training and validation.
Population characteristics	Data for this study was derived from 1979 children (aged 6–12 years), who attended the visit in 2001 (baseline), 2002 (1-year follow up) and 2006 (5-year follow up) visits in the Singapore Cohort Study of the Risk Factors of Myopia (SCORM) study.
Recruitment	Singapore Cohort Study of the Risk Factors of Myopia (SCORM) study, 1979 schoolchildren in grades 1 to 3 (ages 7–9 years) attending three elementary schools in Singapore were recruited from 1999-2001. Seven annual follow-up visits were conducted in the schools.
Ethics oversight	The study was approved by the Ethics Committee at the Singapore Eye Research Institute and the Centralized Institutional Review Boards of the Singapore Health Services (2016/2215) and conducted in accordance with the tenets of the Declaration of Helsinki.

Note that full information on the approval of the study protocol must also be provided in the manuscript.

Field-specific reporting

Please select the one below that is the best fit for your research. If you are not sure, read the appropriate sections before making your selection.

☒ Life sciences ☐ Behavioural & social sciences ☐ Ecological, evolutionary & environmental sciences

For a reference copy of the document with all sections, see nature.com/documents/nr-reporting-summary-flat.pdf

Life sciences study design

All studies must disclose on these points even when the disclosure is negative.

Sample size	Sample size calculation is not relevant to this study as the algorithms were trained using data from the Singapore Cohort Study of the Risk Factors of Myopia (SCORM) study that was collected in 1999-2001.
Data exclusions	The primary dataset comprised of 1666 subjects from schools 2 and 3, of which 701 subjects were excluded due to baseline high myopia and/or missing data (clinical/fundus image). The independent test dataset comprised of 313 subjects from schools 1, of which 214 subjects were excluded due to baseline high myopia and/or missing data (clinical/fundus image).
Replication	From a total of 965 children (1878 eyes) with 7456 retina images from school 2 and 3, the deep learning model was trained with 5-fold cross validation for detection of high myopia using 769 children (1502 eyes) with 5945 retina images and tested on 196 children (376 eyes) with 1511 retina images. The models were externally validated on the test dataset using 99 children (189 eyes) with 821 retina images from school 1.
Randomization	Randomization is not relevant to this study as the algorithms were trained using data from the Singapore Cohort Study of the Risk Factors of Myopia (SCORM) study that was collected in 1999-2001.
Blinding	Blinding is not relevant to this study as the algorithms were trained using data from the Singapore Cohort Study of the Risk Factors of Myopia (SCORM) study that was collected in 1999-2001.

Reporting for specific materials, systems and methods

We require information from authors about some types of materials, experimental systems and methods used in many studies. Here, indicate whether each material, system or method listed is relevant to your study. If you are not sure if a list item applies to your research, read the appropriate section before selecting a response.

Materials & experimental systems

n/a	Involved in the study
☒	☐ Antibodies
☒	☐ Eukaryotic cell lines
☒	☐ Palaeontology and archaeology
☒	☐ Animals and other organisms
☒	☐ Clinical data
☒	☐ Dual use research of concern

Methods

n/a	Involved in the study
☒	☐ ChIP-seq
☒	☐ Flow cytometry
☒	☐ MRI-based neuroimaging
