# Supplementary material for: Deep learning system to predict the 5-year risk of high myopia using fundus imaging in children
Source: NPJ Digit Med. 2023 Jan 26;6:10. doi: 10.1038/s41746-023-00752-8 (PMC9879938; doi:10.1038/s41746-023-00752-8)
Supplement: Supplementary file 2 — Supplementary Material [file 41746_2023_752_MOESM2_ESM.pdf]

Supplementary Table 1: Demographics, myopic spherical equivalent (SE) and axial length (AL) status of subjects in the primary and testing dataset, at baseline and in 5 years used in internal and external validation

**Validation**	**Internal Validation**		**External Validation**
**School**	**School 2 & 3**		**School 1**
**Demographics**	**Training With 5-Fold Cross Validation Dataset (80%)**	**Testing Dataset (20%)**	**Testing Dataset**
**No. of fundus images**	5945	1511	821
**No. of eyes**	1502	376	189
**No. of subjects**	769	196	99
**Age, mean (SD), y**	8.67 (1.23)	8.70 (1.18)	9.46 (0.50)
**Males, No. (%)**	375 (48.76%)	97 (49.49%)	50 (50.51%)
**Race, No. (%)**			
**Chinese [1]**	521 (67.75%)	129 (65.82%)	99 (100.00%)
**Malay [2]**	177 (23.02%)	49 (25.00%)	0 (0.00%)
**Indian [3]**	60 (7.80%)	18 (9.18%)	0 (0.00%)
**Eurasian [4]**	3 (0.39%)	0 (0.00%)	0 (0.00%)
**Others [5]**	8 (1.04%)	0 (0.00%)	0 (0.00%)
**Baseline SE**			
**No myopia, eyes (%)**	911 (60.65%)	234 (62.23%)	64 (33.86%)
**Low myopia, eyes (%)**	448 (29.83%)	108 (28.72%)	70 (37.04%)
**Moderate myopia, eyes (%)**	143 (9.52%)	34 (9.04%)	55 (29.10%)
**High myopia, eyes (%)**	0 (0.00%)	0 (0.00%)	0 (0.00%)
**5th year SE**			
**No myopia, eyes (%)**	477 (31.76%)	149 (39.63%)	44 (23.28%)
**Low myopia, eyes (%)**	554 (36.88%)	117 (31.12%)	64 (33.86%)
**Moderate myopia, eyes (%)**	402 (26.76%)	97 (25.80%)	65 (34.39%)
**High myopia, eyes (%)**	69 (4.59%)	13 (3.46%)	16 (8.47%)
**Baseline AL**			
**AL < 26.5mm, eyes (%)**	1502 (100.00%)	376 (100.00%)	189 (100.00%)
**AL ≥ 26.5mm, eyes (%)**	0 (0.00%)	0 (0.00%)	0 (0.00%)
**5th year AL**			
**AL < 26.5mm, eyes (%)**	1474 (98.14%)	368 (97.87%)	173 (91.53%)
**AL ≥ 26.5mm, eyes (%)**	28 (1.86%)	8 (2.13%)	16 (8.47%)

Supplementary Table 2: Comparison of baseline characteristics of subjects from School 1 (external validation) with Schools 2 and 3 (internal validation) using analysis of variance (ANOVA) and Chi-squared test for continuous and categorical variables, respectively

**Baseline Characteristics**	**School**			**P value**	**Bonferroni adjusted P value**	
	**1 (n = 99)**	**2 (n = 397)**	**3 (n = 568)**		School 1 vs. 2	School 1 vs. 3
**Age, mean (SD), y**	9.46 (0.50)	9.70 (0.84)	7.96 (0.87)	< 0.0001	0.020	< 0.0001
**Males, No. (%)**	50 (50.51%)	176 (44.33%)	296 (52.11%)	0.056	-	
**Race, No. (%)**				<0.0001	< 0.0001	< 0.0001
**Chinese**	99 (100.00%)	230 (57.93%)	420 (73.94%)			
**Malay**	0 (0.00%)	138 (34.76%)	88 (15.49%)			
**Indian**	0 (0.00%)	22 (5.54%)	56 (9.86%)			
**Others**	0 (0.00%)	7 (1.76%)	4 (0.71%)			
**Baseline SE**				<0.0001	< 0.0001	< 0.0001
**No myopia, eyes (%)**	64 (33.86%)	379 (49.03%)	766 (69.32%)			
**Low myopia, eyes (%)**	70 (37.04%)	298 (38.55%)	258 (23.35%)			
**Moderate myopia, eyes (%)**	55 (29.10%)	96 (12.42%)	81 (7.33%)			
**High myopia, eyes (%)**	0 (0.00%)	0 (0.00%)	0 (0.00%)			

Supplementary Table 3: Table of comparison of our AI models with others currently available in the medical literature

Study	Aims	Design of Study	Subjects	SE Prediction			Our Paper	
				**Outcome**	**Input**	**AUC**	**AUC**	**Justification**
Chen et al (2019)<br><br>Contribution of Genome-Wide Significant Single Nucleotide Polymorphisms in Myopia Prediction: Findings from a 10-year Cohort of Chinese Twin Children.	To determine the added predictive ability of genome-wide significant single nucleotide polymorphisms (SNPs) in refraction prediction in children and investigate the earliest age threshold for an accurate prediction of high myopia.	Prospective longitudinal study	1063 first-born twins followed annually between 2006 and 2015 in China<br><br>aged 7-15<br>(10.5 ± 2.2) years old, twins	SE ≤-6.00 at 18 years old from:<br><br>All baseline participants	Age, Age-sqaure, Gender, Baseline SE*	0.96	0.96 (internal validation)<br>0.94 (external validation)<br><br>0.97† (internal validation)<br>0.93† (external validation)	• Our models predicted high myopia in younger group of children aged 6-12 years old, a critical time period to institute timely myopia control treatment to limit high myopia with comparable performance.<br>• Our image model (AUC 0.94) can eliminate the need for cycloplegic refraction in prediction, using only fundus image alone.
				Participants with 1 follow-up visit	Age, Age-square, Gender, Baseline SE* + 1 follow up SE*	0.97	0.98 (internal validation)<br>0.96 (external validation)<br><br>0.99† (internal validation)<br>0.95† (external validation)	
				Participants with at least 2 follow-up visits	Age, Age-square, Gender, Baseline SE* + 2 follow up SE*	0.98	NA	
Lin et al (2018)<br><br>Prediction of myopia development among Chinese school-aged children using refraction data from electronic medical records: A retrospective, multicentre machine learning study.	To apply big data and machine learning technology to develop an algorithm that can predict the onset of high myopia, at specific future time points, among Chinese school-aged children.	Retrospective, multicentre machine learning Study	687,063 multiple visit records (≥3 records) of 129,242 individuals from 8 ophthalmic-centre-based electronic medical record databases between January 1, 2005 and December 30, 2015 and 17,113 follow-up records of 3,215 participants in population-based cohorts.<br><br>aged (8.1 ± 1.5) years old	SE ≤-6.00 in 5 years	Age + 2 or more annual SE* progression	0.88-0.90 (internal validation)<br>0.85-0.92 (external validation)	NA	• With baseline fundus image alone, we were able to achieve an AUC of 0.94, without the need for cycloplegic refraction and a minimum of 3 annual visits (2 or more annual progression) for prediction.
				SE ≤-6.00 at 18 years old in 5 years	Age + 2 or more annual SE* progression	0.86-0.90		

*Spherical equivalent (SE) was performed using cycloplegic autorefraction
†Additional variable, race was also included

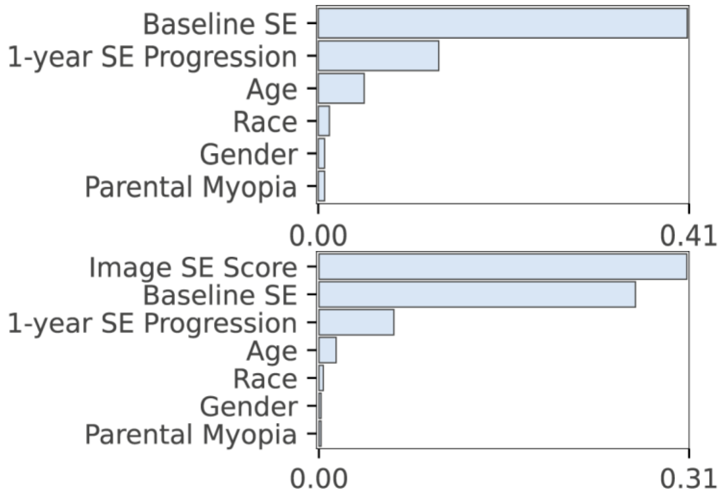

Supplementary Figure 1: Random Forest feature importance output for clinical models (top) and mixed models (bottom)
